# Cement Industry Pollution Mitigation: A Comprehensive Review on Reducing Environmental and Health Impacts

**DOI:** 10.3390/toxics14020138

**Published:** 2026-01-30

**Authors:** Kamal Hosen, Alina Bărbulescu

**Affiliations:** 1 Department of Mechanics, Materials and Structures, Budapest University of Technology and Economics, 1111 Budapest, Hungary; kamalhosen@edu.bme.hu; 2 Department of Civil Engineering, Transilvania University of Brasov, 500152 Brasov, Romania

**Keywords:** cement production, pollution mitigation, carbon capture, alternative fuels, water treatment, noise pollution, soil contamination, sustainable development, emission reduction, waste management

## Abstract

Cement production exerts a significant negative impact on the environment through the emission of greenhouse gases, particulate matter (PM), heavy metals, and other toxic substances into the atmosphere, soil, and bodies of water, degrading the environment and affecting the population’s health. This study reviews different solutions to reduce pollution and mitigate its effects. Particular attention is given to Carbon Capture, Utilization, and Storage (CCUS) technologies and their ability to significantly reduce CO_2_. Biomass and waste-derived fuels were identified as viable substitutes for fossil fuels, although challenges related to supply chain reliability and secondary environmental impacts remain. The study further examined mitigation strategies for non-gaseous pollutants, including noise pollution control measures such as sound barriers and vibration isolation systems, soil remediation techniques such as phytoremediation and the recycling of cement kiln dust (CKD), and water pollution control technologies, including filtration, chemical precipitation, biological treatment, and Zero Liquid Discharge (ZLD) systems. Key research gaps were identified, particularly concerning the long-term durability, scalability, and cost-effectiveness of these mitigation approaches. Overall, the review emphasizes the need for integrated pollution control strategies to support the transition toward a more sustainable cement industry and recommends future research focused on developing mitigation technologies that are efficient, economically viable, and adaptable to large-scale industrial applications.

## 1. Introduction

As one of the world’s most energy-intensive manufacturing sectors, the cement industry is a primary driver of global environmental degradation, with far-reaching consequences for both ecosystems and human health [1,2,3,4]. The core of this impact lies in the production of clinker; the calcination of limestone alone accounts for approximately 5–7% of global carbon dioxide (CO_2_) emissions [5,6,7]. Beyond carbon, the manufacturing process releases substantial quantities of nitrogen oxides (NO_x_), sulfur dioxide (SO_2_), particulate matter (PM), and heavy metals, which contaminate the air, soil, and water, posing severe risks to the surrounding population [7,8].

The sector’s contribution to air pollution is pervasive, as CO_2_ is generated at every stage of clinker production—both through the chemical decomposition of calcium carbonate and the combustion of fossil fuels required to reach high kiln temperatures. This combustion process is also the primary source of NO_x_ emissions, while the oxidation of sulfur compounds within the raw materials and fuels generates SO_2_. When released into the atmosphere, these gases act as precursors to acid precipitation, which further degrades local biodiversity and infrastructure [9,10]. Furthermore, particulate matter (PM) represents a critical operational challenge; fugitive dust generated during the extraction, transportation, and handling of raw materials accounts for more than 90% of the total emissions from cement facilities [11,12,13,14,15].

In addition to the challenges of fuel combustion and greenhouse gas (GHG) emissions, there is an increasing focus—particularly in urbanized areas—on the noise pollution generated by cement plant operations [7]. The continuous operation of heavy machinery, such as crushers, kilns, and grinding mills, produces high-decibel acoustic environments that can result in significant auditory impairment for workers, including noise-induced hearing loss. Beyond physical damage, persistent exposure to these noise levels is linked to broader detrimental effects on human health, such as heightened stress, hypertension, and cognitive fatigue, necessitating more robust mitigation strategies within the industry. The noise created by cement industry operations harms nearby residents as well if there are not adequate noise control measures in place [16,17,18,19,20,21,22,23].

Soil and groundwater contamination caused by Cement Kiln Dust (CKD) represents another critical environmental concern within the industry. As a byproduct of the clinkerization process, CKD is characterized by its high alkalinity and significant concentrations of heavy metals [24,25,26]. When management practices are inadequate, this fine particulate matter can migrate from storage sites and settle on surrounding landscapes [27]. Wimalawansa and Wimalawansa [28] highlight that such mismanagement allows CKD to leach into the soil profile and infiltrate local aquifers, leading to long-term degradation of land quality and the contamination of essential water resources in the vicinity of manufacturing facilities.

Water pollution stemming from cement manufacturing has long been identified as a critical environmental challenge, primarily due to the industry’s intensive water requirements for cooling systems and raw material processing [29,30]. When industrial wastewater is discharged into local water bodies such as lakes and rivers, it carries a heavy load of suspended solids, dissolved salts, and heavy metals such as zinc (Zn) and lead (Pb) that can significantly exceed safe regulatory limits [31,32,33]. These contaminants not only degrade the physical and chemical quality of the water, increasing turbidity and altering pH levels [34,35], but also pose a persistent threat to aquatic biodiversity. The accumulation of these toxic substances can lead to bioaccumulation within the food chain, resulting in long-term disruption to aquatic ecosystems and potentially impairing water resources used for domestic and agricultural purposes in the surrounding regions.

Table 1 summarizes the main pollutants from cement plants and identifies their primary sources.

The environmental repercussions of cement production do not manifest as isolated phenomena within discrete ecological compartments; rather, the pathways to pollution are intrinsically linked through complex biogeochemical interdependencies. Airborne emissions (e.g., particulate matter (PM), nitrogen oxides (NO_x_), sulfur dioxide (SO_2_), and trace heavy metals) are subject to atmospheric deposition, whereby they transition from gaseous or aerosol phases into terrestrial and aquatic sinks. This depositional flux initiates secondary contamination cycles, altering soil chemistry and water quality far beyond the point of emission.

Furthermore, the mismanagement of industrial by-products, specifically Cement Kiln Dust (CKD), represents a significant risk to environmental integrity. When stored or disposed of improperly, CKD is susceptible to chemical leaching and physical mobilization by stormwater. These hydro-geological processes serve as vectors, transporting concentrated pollutants from stabilized solid waste into mobile phases that infiltrate groundwater and surface-water systems. Consequently, the dynamic interaction between precipitation and solid waste creates a recurring source of terrestrial and aquatic degradation.

Adopting a multi-media analytical framework—evaluating air, land, and water simultaneously—is therefore essential for robust, comprehensive environmental regulatory oversight. This systemic approach allows for the identification of “co-benefits,” where technological interventions aimed at primary emission reduction simultaneously yield positive externalities. For example, optimizations in energy efficiency and kiln operations not only mitigate carbon intensity but also reduce acoustic pollution and improve the occupational and public health outcomes for both employees and surrounding communities. Given the projected growth of cement demand in developing economies, there is an urgent need to develop and implement effective strategies to mitigate these harmful effects and align the industry with the SDGs [29,34,36].

The primary objective of this review is to evaluate the current state of technology used to mitigate pollution from the cement industry. By identifying significant technical advancements alongside the practical obstacles that hinder the transition to environmentally friendly production, this study seeks to provide an examination of existing pollution control techniques while highlighting critical research gaps and technical needs essential for supporting sustainable manufacturing practices in the cement sector. To address these complex challenges, the article provides an overview of decarbonization strategies and evaluates the efficacy of specific interventions, including noise attenuation measures, soil remediation strategies, and the advanced management of industrial wastewater and urban runoff.

Beyond the technical assessment, the study seek at identifying the socio-technical barriers to implementation and propose policy frameworks necessary to institutionalize the co-beneficial management of multiple pollutants. By clarifying the balance between different environmental factors, this review aims to empower stakeholders with the insights required to implement more resilient, environmental governance programs that harmonize industrial output with ecological preservation.

## 2. Strategies for Reducing CO_2_ Emissions from Cement Manufacturing

**Figure 1:**
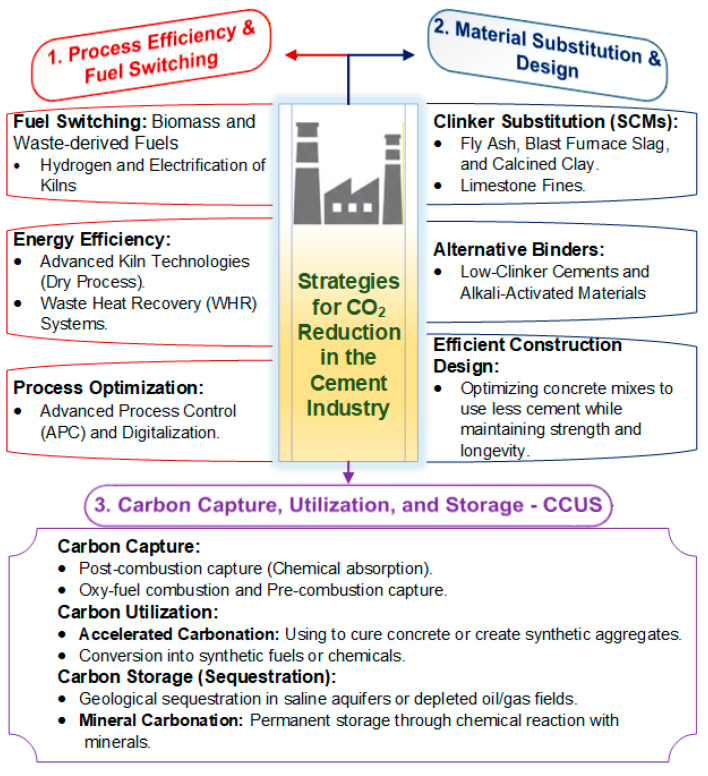
CO_2_ emission reduction strategies in cement production.

The most significant challenge in decarbonizing the cement industry is the reduction in CO_2_, the primary driver of anthropogenic climate change. Given that CO_2_ emissions are inherent to both the chemical decomposition of limestone and the high-temperature combustion of fuels, developing effective mitigation strategies requires a multifaceted approach [37,38]. Consequently, current research has yielded a diverse range of technological pathways, varying from the adoption of alternative raw materials to the implementation of advanced carbon capture systems, each presenting unique trade-offs in efficiency and industrial scalability. Figure 1 shows the main strategies for reducing CO_2_ emissions in cement production, including carbon capture and energy efficiency.

### 2.1. Carbon Capture, Utilization, and Storage Technologies

Carbon Capture, Utilization, and Storage (CCUS) is among the most studied strategies for mitigating CO_2_ emissions within the cement sector, involving the capture of flue gas emissions for subsequent industrial use or geological sequestration [39,40]. Numerous studies have demonstrated that post-combustion capture technologies can drastically reduce the carbon footprint of cement facilities [41,42,43,44,45,46]. While sequestration remains a vital component of this framework, the industry is increasingly looking toward valorization to offset costs. For instance, Mikhelkis and Govindarajan [47] investigated the efficacy of capturing CO_2_ directly from kiln exhaust before transporting it to underground storage sites. Within this domain, the use of chemical solvents—most notably amines—has proven highly effective for CO_2_ absorption [41,48,49,50,51]. However, the widespread deployment of solvent-based capture is hindered by two primary challenges: the substantial energy penalty required for solvent regeneration and operational vulnerabilities, including solvent degradation over time and the accelerated corrosion of the containment infrastructure [52,53].

Despite its potential, the high energy requirements and associated financial costs of carbon capture technology remain significant barriers to large-scale industrial deployment [54]. Consequently, the cement industry must prioritize research aimed at enhancing capture efficiency while reducing capital and operational expenditures [55,56].

An alternative under investigation is oxy-fuel combustion, a process in which fossil fuels are burned in a concentrated oxygen environment rather than ambient air [57]. This method produces a flue gas stream composed almost exclusively of CO_2_ and water vapor, simplifying the separation process. However, the production of high-purity oxygen is energy-intensive, presenting a major obstacle to commercialization [58,59,60].

Carrasco-Maldonado et al. [61] further explored the transition from conventional air-fired combustion to oxy-combustion systems. Their study compared the feasibility of partial CO_2_ capture (60–75%) versus full capture (90–99%), noting that implementation would necessitate significant structural modifications to existing plant components, such as the calciner and the rotary kiln. While their research confirms that oxy-fuel combustion substantially enhances capture potential, it also identifies critical drawbacks: increased electrical energy consumption, operational complexities related to preventing atmospheric air infiltration, and potential risks to clinker quality due to the altered thermal environment of the kiln.

To facilitate the deployment of CCUS within China’s expansive cement sector, Wang et al. [62] developed a source-sink matching model. This approach evaluates the spatial relationship between carbon emission sources and viable geological storage locations while accounting for the carbon-energy-water nexus. Their findings suggest that implementing CCUS across various cement facilities, depending on the mitigation scenario, could achieve CO_2_ reductions between 238.2 and 715.0 million tons. However, the associated costs are substantial, ranging from 79.4 billion to 269.5 billion CNY, and successful implementation remains contingent upon the local availability of sufficient water and energy resources [63].

Beyond storage options, there are numerous ways to utilize the captured CO_2_ to generate environmental benefits and economic value. For example, CO_2_ can be used to produce synthetic carbonates for building materials or integrated directly into concrete production. In this process, the carbon dioxide is mineralized and incorporated into concrete, resulting in a stronger product while simultaneously reducing the carbon footprint of the cement industry. Currently, several industry leaders are adopting these mineralization technologies during the manufacturing phase to create more sustainable building products [64]. By integrating directly into the mixing cycle, these methods lower the net emissions associated with the production of infrastructure. Another significant application is Enhanced Oil Recovery (EOR). In this method, captured is injected into existing oil reservoirs to increase the volume of crude oil that can be extracted. EOR represents one of the most established commercial uses for captured carbon, creating a strategic avenue where industrial emissions can be repurposed to benefit both the energy and cement sectors through a circular economy framework [65,66,67]. The success of these utilization and storage pathways, however, depends heavily on the efficiency of the initial capture stage at the plant.

Despite these possibilities, large-scale utilization is currently constrained by economic feasibility and the logistical gap between current usage rates and the massive volume of CO_2_ generated by the industry [56]. While long-term sequestration in depleted oil fields and saline aquifers has been proven to reduce atmospheric CO_2_ [68,69], Udebhulu et al. [70] emphasize persistent concerns regarding potential leakage and the long-term structural integrity of these storage sites. To avoid storage risks, Meijssen et al. [71] proposed an Integrated Carbon Capture and Utilization (I-CCU) approach. Their model analyzes the conversion of captured CO_2_ into methanol using hydrogen produced via electrolysis. By comparing this to Business-as-Usual (BAU) and standard Carbon Capture and Storage (CCS) methodologies, the study highlights that while I-CCU offers a circular carbon economy, the extreme energy demands and economic costs associated with electrolysis and capture remain significant barriers to widespread industrial adoption.

Several studies [72,73,74] have conducted cost analyses of carbon capture, utilization, and storage (CCUS) technologies. Zhou et al. [72] focus on optimizing CCUS systems in Shanxi Province using a mixed-integer linear programming (MILP) model to minimize the total annual costs (TAC) associated with CO_2_ capture, transportation, sequestration, and utilization. Their results indicate that CO_2_ utilization for urea production is the most economically viable option, achieving over 50% emissions reduction at the lowest cost. CO_2_-enhanced energy-efficient concrete blocks (CO_2_-EECB) and cement curing were also found to be economically feasible, whereas microalgae cultivation was identified as the least cost-effective option.

Miao et al. [73] evaluate the economic performance of China’s first million-ton CCUS-enhanced oil recovery (CCUS-EOR) project by analyzing its net present value (NPV), energy return on investment (EROI), and carbon return on investment (CROI). The project achieved an NPV of $56.09 million, an internal rate of return (IRR) of 13.4%, and a payback period of 8.34 years, indicating overall economic viability. However, the authors note that profitability is sensitive to oil price fluctuations, with an economic viability threshold of $81.52 per barrel.

In contrast, Lin et al. [74] adopt a broader perspective on CCUS within the context of China’s decarbonization strategy, particularly in the steel industry. They emphasize CCUS as a critical component of China’s low-carbon transition but highlight the substantial capital, operational, and maintenance costs associated with large-scale deployment. Unlike Zhou et al. [72] and Miao et al. [73], who identify specific CCUS technologies and projects that can be economically viable, Lin et al. [74] argue that widespread industrial adoption will require technological innovation, expanded infrastructure, and a comprehensive policy framework supported by government intervention and market mechanisms. All these studies underscore both the opportunities and challenges associated with deploying CCUS technologies for industrial CO_2_ mitigation, emphasizing the need to improve cost-effectiveness and ensure long-term financial sustainability.

### 2.2. Use of Alternative Fuels

The use of alternative fuels is recognized as an effective approach for reducing the greenhouse gas emissions from cement manufacturing, often complementing Carbon Capture technologies [75,76,77,78,79,80]. Traditionally dependent on coal and other fossil fuels, this industry is increasingly transitioning toward biomass, waste-derived fuels, and by-products from industrial manufacturing to mitigate its carbon footprint [78,79,80,81,82].

Biomass is considered a carbon-neutral energy source [83,84,85,86] as the CO_2_ released during combustion is theoretically offset by the carbon sequestered during the plant’s growth cycle. Therefore, several studies analyzed its use as a substitute fuel in cement manufacturing, emphasizing its potential to decrease CO_2_ emissions. For instance, Salaripoor et al. [80] replaced fossil fuels with biomass and waste-derived fuels in cement production and proved that the carbon footprint decreased.

Beyond direct combustion, the chemical properties of biomass ash offer further opportunities for decarbonization. Tosti et al. [78] evaluated biomass as a raw material substitute in clinker production, finding that wood biomass with high bark content produces ash with optimal properties for clinker formation. Despite these benefits, biomass supply chains remain a challenge, requiring consistent feedstock availability and pre-treatment to ensure combustion stability [86]. As noted by Lewandowski [87] and Ahorsu et al. [88], the sustainability and scalability of biomass are often compromised by its competition with food production and the high land-use requirements for bioenergy.

Waste-derived fuels, including Refuse-Derived Fuel (RDF), Tire-Derived Fuel (TDF), sewage sludge (SS), and plastics, present a dual solution for fossil fuel replacement and municipal waste management. Ige and Kabeya [89] highlight that RDF and TDF are particularly effective due to their high calorific values and combustion stability, with RDF capable of achieving thermal substitution rates exceeding 80%. In contrast, biomass and sewage sludge often require drying or pelletization to improve their energy density and performance within the kiln. Environmental assessments suggest that optimizing the use of these waste-derived fuels can reduce CO_2_ emissions by 15% to 30%, while simultaneously decreasing SO_x_ and NO_x_ levels. Nevertheless, significant obstacles persist: (a) Variability in moisture content and chemical composition can lead to unstable kiln temperatures and affect clinker quality. (b) The combustion of certain waste materials and biomass can introduce heavy metals and dioxins into the process, necessitating rigorous emissions monitoring and advanced filtration to protect public health [90,91]. High costs, regional availability of feedstocks, and varying environmental regulations limit the widespread adoption of these fuels for large-scale facilities [14,92]. For instance, biomass fuels may not be available in sufficient quantities to meet the needs of large cement manufacturing facilities, and the use of waste-derived fuels may create problems related to the combustion of hazardous materials.

Fang et al. [93] and Maschowski et al. [94] investigate promising alternatives to conventional cement production with the aim of maintaining comparable material quality and processing standards while reducing environmental impacts through the use of nontraditional fuels and raw materials. Fang et al. [93] demonstrate the successful use of sewage sludge as a secondary fuel and as a source for NO_x_ denitration, achieving a significant reduction in NO_x_ emissions without affecting cement quality. However, they observed a slight delay in cement setting time, attributed to the presence of trace elements in the sludge.

Similarly, Maschowski et al. [94] assess alternative materials with a particular focus on biomass ashes derived from the combustion of wood chips and Miscanthus straw. Their results indicate that both bottom ash and Miscanthus ash enhance cement flowability and compressive strength. In contrast, ashes collected from electrostatic precipitators and baghouse filters exhibited substantially lower strength due to high concentrations of soluble potassium salts, which increased water demand. A key outcome is the importance of understanding ash composition, particularly the presence of trace metals, Maschowski et al. [94] specifically identifying zinc and cadmium as contaminants that are immobilized in the cement matrix. While Fang et al. [93] emphasize the environmental benefits associated with NO_x_ reduction, Maschowski et al. [94] emphasize the environmental benefits associated with NO_x_ reduction, focus more on optimizing biomass ash characteristics to improve cement performance. Overall, both studies highlight the critical role of pre-treatment and stringent quality control when incorporating alternative materials into cement production.

### 2.3. Cleaner Production Technologies

Although alternative fuels and materials can substantially reduce greenhouse gas emissions, achieving long-term sustainability necessitates the adoption of Cleaner Production (CP) practices that optimize efficiency across the entire cement manufacturing chain, from raw material extraction to final clinker cooling.

One of the most effective strategies for reducing the sector’s carbon intensity is the partial substitution of conventional clinker with Supplementary Cementitious Materials (SCMs), such as fly ash, blast-furnace slag, and calcined clays [95,96]. By reducing the clinker-to-cement ratio, these SCMs significantly reduce process-related CO_2_ emissions arising from limestone calcination. While several studies have shown that blended cements can maintain, and in some cases exceed, the mechanical strength and durability of traditional Portland cement [97,98], others emphasize constraints related to regional availability of suitable materials and potential variability in chemical composition, which can affect product consistency.

A separate body of literature focuses on energy-efficiency measures that are essential for decarbonization rather than material substitution. Schneider et al. [99] highlight that given the inherently energy-intensive nature of cement manufacturing, maximizing heat recovery offers a more universally applicable mitigation strategy. Waste Heat Recovery (WHR) systems capture thermal energy from kiln exhaust and clinker cooler gas streams to generate electricity, thereby reducing dependence on external power supplies [100]. In a technical evaluation of WHR efficacy, Fierro et al. [101] simulated various Organic Rankine Cycle (ORC) systems and drying units, demonstrating that toluene provides superior thermal-to-electric conversion efficiency relative to other working fluids. Although these studies acknowledge the high capital investment required, economic assessments suggest that the long-term reduction in energy costs and CO_2_ emissions justifies the investment, particularly as the industry moves toward global decarbonization targets.

The integration of renewable energy sources, such as wind and solar, to power plant infrastructure offers an additional pathway to decouple the cement industry from fossil fuel dependence [9]. Akintayo et al. [102] show that combining material substitution (e.g., clinker with waste-derived materials) with renewable electricity sources, such as wind and solar power, can reduce energy consumption up to 44% and CO_2_ emissions by 50%.

However, unlike model-based analyses that assume optimal conditions, empirical assessments stress that the commercial viability of such integrated approaches is highly dependent on plant-specific configurations and local energy resource availability [9]. As a result, the literature collectively underscores that no single mitigation pathway is universally optimal, reinforcing the need for context-specific, flexible strategies supported by continued research and development [103].

### 2.4. Transition to Green Cement

A transformative approach to lowering the sector’s carbon footprint is the transition to ‘Green Cement,’ characterized by the use of low-energy production techniques and alternative raw materials [104]. A significant portion of these advancements focuses on alternative binders (e.g., geopolymers) and alkali-activated cements, which decouple cement production from the carbon-intensive calcination of limestone [105,106].

Various green cement technologies have been implemented in commercial production. One example of this is a company. One such example involves a process in which carbon dioxide captured from industrial emissions is chemically converted into a binding material that permanently encapsulates the sequestered carbon. Another emerging technology is geopolymer cement which is produced using various industrial by-product materials (such as slag or fly ash) and is now being used in construction projects aimed at reducing the carbon footprint of building materials. Despite these advancements, the adoption of green cement products remains limited compared to traditional Portland cement due to higher production costs and underdeveloped infrastructure. Nevertheless, demand for low-carbon alternatives is increasing, particularly in regions with stringent environmental regulations or incentives for sustainable building practices. Many certified projects and other sustainable construction efforts are incorporating green cement products to meet environmental performance goals. However, conventional cement continues to dominate the marketplace because of its greater cost efficiency and well-established supply chain [107,108,109,110].

In an effort to identify low-cost solutions that would help in reducing CO_2_ emissions associated with the manufacture of conventional Portland cements, Gartner and Sui [111] conducted extensive research to identify various alternative cement clinker compositions and curing approaches. Their analysis identified several promising pathways with varying mitigation potential. Belite–Ye’elimite–Ferrite (BYF) cements achieved an estimated 24% reduction in CO_2_ emissions through reduced limestone demand and lower kiln temperatures, while Reactive Belite-rich Portland Cement (RBPC) offered a more incremental 10% reduction within a framework compatible with existing Portland cement infrastructure. More substantial emission reductions were observed for Calcium Cold-Sintered Cement (CCSC), which demonstrated up to a 37% CO_2_ decrease by employing carbonation-based curing processes that actively consume CO_2_. Magnesium Oxide–based systems (MOMS) were identified as the most transformative option, with the potential to produce carbon-negative concrete by sequestering more CO_2_ over their lifecycle than is emitted during production.

Complementing this assessment, Juenger et al. [112] critically examined the technical and economic limitations affecting the practical deployment of several alternative binders, including Calcium Aluminate Cement (CAC), Calcium Sulfoaluminate Cement (CSA), supersulfated cements, and geopolymer binders. Although CAC provides rapid early strength, its susceptibility to mineralogical “conversion” under humid or weathering conditions can result in significant long-term strength loss. CSAs exhibit favorable durability and a comparatively low carbon footprint, yet their dependence on bauxite renders them costly and limits large-scale adoption. Geopolymer binders derived from industrial by-products such as fly ash and slag offer strong potential for circular-economy integration and deep decarbonization; however, their commercial viability is currently constrained by high production costs and logistical challenges associated with scaling liquid-activator systems. While these technologies remain at an early stage of commercialization, their capacity to substantially decarbonize the cement industry is widely recognized [113]. At present, many manufacturers are exploring these alternative binders in parallel with renewable energy adoption and waste-derived fuels, but widespread implementation continues to be hindered by high capital investment requirements and unresolved technological barriers.

## 3. Strategies for Reducing Noise Pollution in Cement Manufacturing

Although CO_2_ emissions are the primary target of decarbonisation strategies in the cement industry, cement manufacturing also generates a spectrum of local environmental pollutants that are equally relevant to environmental quality and public health. Emissions into air (including dust and atmospheric toxins), discharges affecting water and soil, and physical disturbances arising from industrial operations collectively shape the environmental burden experienced by communities surrounding cement production facilities. The mitigation measures discussed earlier—such as fuel substitution, process optimisation, and equipment upgrades—therefore influence not only greenhouse gas emissions but also the nature and intensity of these site-level impacts. Within this broader pollution framework, noise pollution represents a critical yet comparatively underexamined exposure pathway. Frequently occurring alongside air, water, and soil contamination, noise contributes to cumulative environmental stress within cement industrial zones. Accordingly, this section transitions from global climate-focused considerations to a site-specific examination of noise pollution as an integral component of the overall environmental footprint of cement manufacturing.

**Figure 2:**
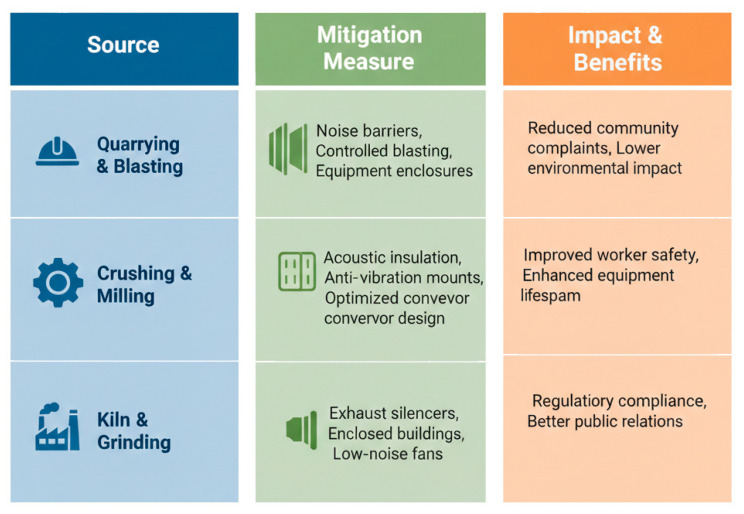
The main noise sources in the cement industry, mitigation measures and the impact and benefit of their implementation.

Figure 2 presents the main noise sources in the cement industry and the strategies for noise reduction.

### 3.1. Technological Solutions for Noise Mitigation

To mitigate the acoustic impacts of cement manufacturing, a combination of sound isolation and absorption, and directional noise-diversion strategies is primarily implemented within production facilities [17]. These measures are designed to protect both on-site workers and surrounding residential communities from the high-decibel environments characteristic of heavy industry. Koonsman [114] conducted a detailed analysis of noise reduction strategies for critical equipment, including crushers, grinding mills, kiln burners, and compressors, which can generate sound pressure levels of up to 106 dBA. The study recommends several proactive engineering controls:

- Source Encapsulation and Damping: Enclosing high-noise machinery in soundproof housings and utilizing rubber liners in ball mills can reduce acoustic output by up to 10 dBA.
- Vibration Control: Structural design plays a vital role in noise suppression; the use of massive, high-inertia foundations is recommended to minimize the mechanical vibrations from large mills that otherwise propagate through the plant floor.
- Operational Insulation: Implementing insulated control rooms and optimizing plant layouts to isolate noise-intensive zones from high-traffic worker areas significantly reduces cumulative exposure.

Regulatory compliance, such as adhering to the 90 dBA limit for continuous 8 h exposure and progressively reducing the working hours as sound levels increase, remains critical in order to prevent permanent noise-induced hearing loss. Engineering controls must be complemented by high-quality personal protective equipment (PPE), providing a last line of defense while also improving community relations through reduced external noise.

Building on these approaches, Kirk [115] focused specifically on vibration isolation systems as a primary solution for attenuating structure-borne noise from heavy machinery. His research demonstrates that isolating the mechanical energy from equipment such as grinding mills, compressors, and industrial fans can substantially disrupt sound transmission. In the specific case of grinding mills, Kirk documented the implementation of acoustical curtains. When correctly installed, these heavy-duty barriers achieved a profound reduction in localized noise exposure, lowering sound pressure levels from a range of 104–112 dBA down to 94–96 dBA. Similar strategies applied to compressors and cooling fans further contributed to quieter operations. These acoustic improvements have direct implications for occupational health. By successfully reducing ambient noise, the plant can extend permissible exposure times (PET) for personnel in accordance with safety standards. Because the relationship between decibel levels and hearing damage is logarithmic, a reduction of even 10 dBA represents a tenfold decrease in sound intensity, allowing workers to perform tasks in these areas for significantly longer intervals without exceeding daily noise dose limits.

Canfeng et al. [17] present a practical demonstration of an integrated noise-mitigation strategy at a dry-process cement facility. Their findings highlight the effectiveness of targeting high-decibel sources, particularly roots blowers, where replacing damaged silencers with high-efficiency units achieved a 25 dB reduction at the source. Secondary noise propagation was mitigated through structural upgrades, including noise-absorbing doors, noise-attenuated windows, and advanced acoustic materials on walls and ceilings, increasing the facility’s average sound absorption coefficient from 0.03 to 0.32. Collectively, these measures reduced average daily noise levels from a hazardous 102.9 dBA to 88.3 dBA, approaching the regulatory threshold of 85 dBA and substantially lowering the risk of noise-induced hearing loss for workers.

The work of Hadži-Nikolova et al. [116] took another approach. They built an acoustic enclosure around a noisy blower to lower the noise from 93.5 dB(A) to 86.9 dB(A). In addition, they replaced noisy fans with quieter versions and installed silencers and acoustic panels around crushers and exhaust fans to help reduce noise. These changes significantly reduced noise levels at the plant, creating a better working environment and lowering the potential negative impact on neighbouring residential neighbourhoods.

To complement these findings, Ali et al. [117] targeted operational processes and personal protective equipment. According to these authors, the noise is predominantly due to aerodynamic noise that occurs during the operation of the rotating machinery and impact noise from materials colliding inside the mills. Therefore, they recommended using regular noise level monitoring programs, balancing rotating equipment, and aligning all elements of a rotary assembly to help minimize exposure to noise, as well as the use of noise barriers, air mufflers, and hearing protection by the employees.

While technological interventions—such as acoustic barriers, specialized silencers, and vibration isolation systems—have proven effective in attenuating industrial noise, their implementation often entails substantial financial investment. The capital expenditure required for high-performance materials and specialized engineering can represent a significant economic barrier for many operators [118,119]. Beyond direct equipment costs, spatial restructuring of plant layouts offers an additional strategy for reducing noise exposure. By strategically increasing the distance between high-decibel equipment and worker stations or nearby residential areas, facilities can exploit the inverse square law of sound propagation to achieve natural reductions in noise levels.

However, the feasibility of such spatial adjustments is often constrained by site-specific limitations, particularly in older, land-constrained facilities or plants located in densely populated urban corridors. As a result, the integration of advanced noise-control technologies requires careful balancing of regulatory compliance, worker safety, and economic viability. These considerations highlight the need for cost-effective, modular, and adaptable acoustic solutions, representing a critical avenue for future research and development within the cement industry.

### 3.2. Administrative Controls, Noise Mapping, and Layout Optimization

Beyond physical barriers, effective mitigation of industrial noise relies heavily on administrative controls and strategic operational design. Zimwara et al. [120] highlight occupational shift rotation as a key strategy for managing cumulative noise exposure. By alternating personnel between high-decibel zones and acoustically quieter areas, operators can significantly reduce the risk of Noise-Induced Hearing Loss (NIHL). Furthermore, scheduling noise-intensive processes, such as primary crushing and clinker grinding, during low-occupancy shifts can minimize the number of employees subjected to peak acoustic levels.

Predictive modeling and visualization further enhance noise management. Prascevic et al. [121] employed advanced noise mapping at the Holcim cement facility in Serbia and modelled the spatial and temporal propagation of noise into surrounding communities. This approach enables the identification of acoustic “hotspots” and the implementation of targeted mitigation measures before noise reaches unacceptable levels.

Optimization of the plant layout provides a cost-effective and proactive approach to noise reduction. Ning et al. [122] developed a Multi-Objective Optimization Model combining Ant Colony Optimization (ACO) and Genetic Algorithms (GA) to determine the optimal placement of temporary and permanent facilities. By maximizing the distance between workers and high-noise machinery, these models enhance both safety and operational efficiency without necessarily increasing capital expenditure.

A robust Hearing Conservation Program (HCP) underpins these administrative and design measures. Such programs rely on the consistent provision of high-attenuation Personal Protective Equipment (PPE), including earplugs and earmuffs, complemented by comprehensive employee training [123,124]. Proper fitment and awareness of the long-term physiological consequences of noise exposure are essential for PPE to be effective.

Despite these measures, administrative and design-based alterations face significant practical hurdles. Strategic scheduling of noise-intensive operations is often restricted by rigid production quotas and the continuous nature of cement manufacturing, which requires 24 h kiln operation. Despite these measures, administrative and design-based noise mitigation strategies face significant practical challenges. Strategic scheduling of noise-intensive operations is often constrained by rigid production quotas and the continuous 24 h operation of cement kilns. Similarly, while layout modifications and engineering controls can provide lasting noise reductions, their implementation depends on available capital and site-specific spatial constraints. Consequently, an effective noise mitigation strategy must balance the ideal of source reduction with the economic and operational realities of modern cement facilities.

Efforts to manage noise often overlap with broader environmental controls, including the installation of sound-absorbing materials, the erection of enclosures or fences, and the regulation of vehicle access to limit both traffic-related noise and fugitive dust emissions. Dust and particulate matter generated during these processes frequently contain toxic trace metals and other contaminants, meaning that controlling dust at the source is not only critical for air quality but also for preventing the transfer of pollutants to surrounding environments. This linkage naturally leads to the next environmental concern: soil pollution. Deposition, accumulation, and persistence of dust and heavy metals from cement manufacturing can contaminate terrestrial environments adjacent to production facilities, making soil management a vital component of integrated pollution control.

## 4. Strategies for Reducing Soil Pollution in Cement Manufacturing

The environmental sustainability of the cement sector is significantly challenged by the emission of Particulate Matter (PM) and Heavy Metals (HMs), which serve as primary vectors for soil degradation. These contaminants, including trace elements such as lead, cadmium, and chromium, alter the chemical composition of the surrounding pedosphere, directly inhibiting plant growth and reducing agricultural productivity [125,126]. Given that HMs are non-biodegradable, they persist in the soil and groundwater for decades, creating a legacy of contamination that poses long-term risks to both local flora and human populations. Research indicates that the concentrations of HMs in the vicinity of cement facilities frequently exceed regulatory thresholds. Once deposited, these metals can enter the food chain through bioaccumulation in crops, leading to chronic health risks for residents in neighboring communities [127]. The transition of these pollutants from atmospheric emission to soil deposition necessitates integrated remediation strategies that address both the source of the particulates and the long-term stabilization of contaminated land.

### 4.1. Technological Solutions for Soil Remediation

To mitigate the ecological impact of cement-related pollutants, several remediation strategies have been developed to remove, neutralize, or immobilize HMs within the soil [34,128]. These techniques can be broadly categorized into co-processing in cement kilns, biological methods, and engineering-based approaches such as soil washing and microbial remediation.

#### 4.1.1. Co-Processing of Contaminated Soil in Cement Kilns

Chang et al. [128] explored a circular economy approach by utilizing HMs-contaminated soil as a partial replacement for raw materials in cement kilns. By substituting traditional raw materials with contaminated soil at levels of 3%, 7.6%, and 10%, the study assessed the potential for chemical immobilization within the clinker matrix. The research found that soil containing Chromium (Cr) acted as a retardant, increasing setting times and decreasing the overall compressive strength of the final cement product. While co-processing can effectively trap metals in a crystalline structure, the high concentration of certain HMs can compromise the structural integrity of the resulting concrete.

In the environmental risk assessment of the co-processing of heavy metal-contaminated soils in cement kilns, we need to consider more than just short term performance of cement. We should develop a pathway-based analysis for cement kiln air emissions, particulates that are associated with the co-processing of heavy metals, for example, through stack emissions and particulate emissions, as well as their contribution to kiln dust (CKD) and other residuals. The semi-volatile nature of some heavy metals will result in partial volatilization at kiln operating temperatures and subsequent recondensation in CKD and/or other particulate matter. Therefore, appropriate air pollution control measures, including full air emissions monitoring systems, need to be utilized to minimize potential airborne emissions during the soil substitution periods. In parallel to air emissions monitoring, it is also important to carry out any leach testing for all clinker/cement/concrete that is to be used for construction (including CKD or other residuals) to determine if the immobilization of the metals will continue to remain stable within the environment and that there will be no delayed effects to soils or groundwater.

Integrating human health risk assessment (HRA) methodologies using the concentrations of heavy metals present and exposure pathways that may exist in the community can assist in the development of a safe rate of substitution for the co-processed soils and a monitoring plan for the use of these materials, especially in developing countries where baseline contamination and ability to enforce regulations are often different.

#### 4.1.2. Fito- and Bio- Remediation

An alternative method for co-processing of contaminated soil in cement kilns is phytoremediation [129,130], which leverages plant physiology to extract, sequester, or stabilize heavy metals in soils [131]. Phytoremediation offers less energy-intensive solutions, though their efficacy remains highly site-specific. Species such as *Helianthus annuus* (sunflower) and *Brassica juncea* (Indian mustard) have demonstrated significant HM removal near cement facilities [132].

Raj et al. [133] evaluated the mercury (Hg) extraction potential of *B. juncea* in various fly ash (FA) and garden soil (GS) mixtures. He found that most Hg was stored in the plant roots, with a Bioconcentration Factor (BCF) ranging from 0.17 to 0.63. The highest remediation rate (2.62 mg Hg per kg of biomass) occurred in a 50% FA mixture, suggesting that excessive contamination levels can eventually inhibit the plant’s biological efficiency.

While phytoremediation is a cost-effective ‘green’ technology, its use is limited by the growth rate of the biomass and the plant saturation limits of the plants. In highly contaminated sites, biological methods may require supplemental chemical soil washing to lower toxicity levels to a range that the plants can survive.

Biological remediation offers a pathway for addressing co-contaminated soils containing both HMs and Polycyclic Aromatic Hydrocarbons (PAHs). While heavy metals like Cd, Cu, and Pb typically inhibit microbial activity and enzymatic function, specific resilient strains can survive and thrive in these environments [134,135]. Bacteria such as Bacillus and Mycobacterium, along with certain fungi, have demonstrated the ability to remediate co-contaminated soils. Liu et al. [135] found that using microbial consortia, specifically plant-associated microbes, can enhance remediation by alleviating metal toxicity through synergetic metabolic pathways. These microbes act as “helpers” that protect the primary remediating organisms from the toxic effects of the heavy metals, allowing for more effective degradation of organic pollutants.

Bioremediation, while environmentally friendly, presents several limitations. Its application is restricted to biodegradable pollutants and is largely ineffective against persistent synthetic compounds. The process is inherently slow and often requires extended periods to achieve significant remediation. Moreover, its efficiency is highly dependent on environmental conditions, including temperature, pH, nutrient availability, and oxygen levels. In some cases, pollutants may undergo incomplete degradation, leading to the formation of potentially toxic intermediates. Monitoring and controlling microbial activity in situ is challenging, and the practical application of bioremediation is limited in large-scale or heavily contaminated sites. Additionally, the introduction of non-native or genetically modified microorganisms may pose ecological risks.

#### 4.1.3. Engineering-Based Remediation

Soil washing [136] utilizes water and aqueous solutions, often containing medium-phosphate chelators, to scrub HMs from the surfaces of soil particles. According to Chiu and Gani [137], an effective remedial strategy for metals such as zinc, lead, cadmium, and mercury requires a dual-stage approach:

- Physical Separation: Techniques such as froth flotation, magnetic separation, and gravity concentration are employed to isolate particulate contaminants based on their physical properties (density and magnetism).
- Chemical Extraction: Following physical separation, chelating agents and acids are used to desorb ionic or adsorbed metal contaminants from the soil matrix.

The authors [136,137] emphasize that combining these approaches is essential for maximizing remediation efficiency, particularly in sites degraded by intensive industrial activities or recycling facilities.

### 4.2. CKD and Valorization in Civil Engineering

The rigorous management of industrial by-products is fundamental to mitigating soil pollution, as the improper disposal of CKD and related residuals can lead to the substantial chemical contamination of surrounding terrestrial ecosystems [138,139]. Although CKD constitutes 15–20% of total cement production, its disposal in conventional landfills presents a complex environmental challenge due to high concentrations of alkalis, sulfates, and chlorides that can destabilize soil chemistry. However, Al-Bakri et al. [139] suggest that CKD should be viewed as a beneficial secondary material that supports industrial sustainability through its versatile application in civil engineering. Their findings indicate that CKD can be effectively utilized for soil stabilization by enhancing compaction and reducing permeability, or as a partial substitute for Ordinary Portland Cement (OPC) at low replacement levels of 5–10% without compromising the mechanical integrity of concrete or mortar. Expanding the same idea, Sharifi Teshnizi et al. [140] investigated the capacity of CKD to alter the physical, chemical, and mechanical properties of loess soil in the Semnan Province of Iran. By incorporating CKD into the soil at mixing ratios between 5% and 20%, the researchers subjected the stabilized matrix to rigorous unconfined compressive strength (UCS), direct shear (DS), and soaking durability testing. The results demonstrated a profound mechanical enhancement after a 28-day curing period, with the stabilized soil exhibiting a maximum UCS approximately 9.9 times greater than that of the untreated soil. This evidence underscores the dual benefit of CKD valorization: it not only prevents the environmental degradation associated with industrial waste disposal but also provides a superior, cost-effective additive for large-scale infrastructure and soil remediation projects.

The integration of CKD into cementitious matrices (e.g., pastes, mortars, and concrete) requires a precise understanding of its chemical influence on hydration and durability. Kunal et al. [141] demonstrated that while the incorporation of CKD at low replacement levels (5–10%) can slightly enhance the mechanical strength of cement mixtures, a critical threshold exists at approximately 15% replacement. Beyond this point, both compressive and tensile strengths significantly decline, a phenomenon primarily attributed to excessive alkali concentrations that disrupt the hydration process and compromise the structural integrity of the matrix.

The most important long-term uncertainty for reusing CKD (Cement Kiln Dust), not only the early-age strength of CKD, is how well chemical stabilisation remains effective with long-term wet-dry cycles and groundwater. CKD has soluble alkalis and free lime. If CKD dissolves and leaches enough to change the chemistry of pore-water, permeability control will reduce, making it harder to achieve durability over time–especially in areas where rainfall is abundant, or water table height changes frequently. Leaching testing (such as TCLP tests) has shown potential release of trace metals, including chromium and barium, from cement/CKD matrices depending on pH and test conditions, which reinforces the need for post-treatment verification instead of assuming that immobilisation will last indefinitely. In addition, many studies have been completed, including CKD in geopolymer/alkali-activated and other solidification-stabilisation systems, whereby these systems typically offer better containment capacity and lower cumulative element release with a long-term leaching scenario compared to traditional matrices [141].

To mitigate these risks, source reduction has emerged as a primary strategy for waste management within the cement industry. Through the implementation of closed-loop processes and the optimization of existing kiln parameters, manufacturers have successfully reduced the volume of CKD and other production by-products [142]. Furthermore, research has indicated that the transition to alternative fuel sources, such as biomass and refuse-derived fuels (RDF), can effectively lower the net production of CKD at the cement plant level [143]. However, the transition toward a fully circular waste model remains complex. While minimizing waste and recycling, CKD offers significant environmental opportunities, their success is heavily contingent upon the financial viability of recycling programs and the market’s capacity to absorb products manufactured from CKD. Without these economic drivers, the disposal of CKD in landfills remains a necessary but high-risk practice, leaving many unresolved questions regarding the development of truly sustainable, long-term disposal options for the global cement manufacturing industry.

## 5. Strategies for Reducing Water Quality Degradation from Cement Manufacturing

Water quality and soil pollution are closely interconnected, particularly around cement plant facilities. Unsettled dust or improperly managed residues, such as CKD and other alkaline materials, can be transported by rain, stormwater runoff, or leachate. When dissolved in water, these substances can raise the pH of surface and groundwater and mobilize heavy metals, posing risks to aquatic ecosystems and downstream drinking water resources. Therefore, effective management of cement-related pollution requires an integrated approach addressing both terrestrial and aquatic pathways. In the following, we discuss various strategies to mitigate the impact of the cement industry on the water resources through wastewater treatment, process water recycling, and runoff control.

As previously established, the environmental footprint of cement production extends significantly into the surface water through the discharge of wastewater containing heavy metals, suspended particulate matter, and various chemical additives that compromise water quality and aquatic biodiversity. These pollutants originate from diverse operational stages, including the mechanical processing of raw materials, high-temperature kiln activities, and unmanaged stormwater runoff that leaches contaminants from the facility’s grounds. Specifically, the water utilized for washing limestone, clay, and sand generates high-turbidity effluents characterized by a dense concentration of fine mineral particles and adsorbed contaminants. Furthermore, the leakage of CKD into these water streams increases the pH level, creating highly alkaline conditions that are toxic to sensitive aquatic organisms. The presence of sulfur, nitrogen, and carbonates in effluent discharges further exacerbates environmental degradation by acting as precursors for acidification and eutrophication, which deplete dissolved oxygen and disrupt the ecological balance of receiving water bodies [12,34,144]. Ultimately, these contamination pathways represent a severe risk to the integrity of local drinking water reservoirs, necessitating the implementation of rigorous treatment and containment strategies to protect both human health and regional biodiversity.

### 5.1. Mechanical Solutions for Water Pollution Mitigation from the Cement Industry

To mitigate the hydrological impact of cement production, an integrated suite of technological solutions has been implemented, primarily targeting the rigorous treatment of industrial effluents, the reduction in discharge volumes, and the prevention of aquatic contamination. Filtration remains one of the fundamental methods for managing particulate-laden wastewater within the sector [145]. However, transitioning from conventional treatment approaches to advanced water recycling systems is essential for long-term operational sustainability. Salman [146] demonstrated this necessity through a comparative assessment of water management practices at Cementra Jordan and Lafarge Cement Jordan. While Cementra Jordan relied predominantly on freshwater inputs without internal reuse, Lafarge Cement Jordan exhibited the effectiveness of integrated treatment trains. The proposed incorporation of sedimentation, dual-media filtration, and reverse osmosis (RO) highlighted how advanced mechanical and membrane-based processes can substantially reduce freshwater consumption while enabling comprehensive industrial wastewater recycling.

Beyond direct water treatment, optimization of fuel sources represents an indirect yet impactful strategy for reducing wastewater generation. Sarc et al. [147] investigated the environmental benefits of utilizing Solid Recovered Fuels (SRFs) derived from the Mechanical–Biological Treatment (MBT) of mixed municipal solid waste in cement kilns. Their study, conducted in Croatia, confirmed that SRFs meet stringent cement industry quality requirements, exhibiting low moisture and ash contents alongside acceptable heavy metal concentrations. The substitution of conventional fossil fuels with SRFs not only reduces greenhouse gas emissions but also contributes to lower net wastewater production, thereby improving the overall hydrological sustainability of cement manufacturing facilities.

In addition to conventional filtration systems, advanced treatment technologies such as ultrafiltration (UF) membranes and electrocoagulation (EC) offer high-efficiency pathways for wastewater purification and reuse. Zhu et al. [148] demonstrated that UF membranes are particularly effective at removing suspended solids, organic contaminants, and heavy metals, achieving reported improvements of up to 70% in treated water quality and significantly reducing pollutant loads in final effluents. Complementing these physical separation techniques, electrocoagulation employs electrical currents to destabilize and remove dissolved metal ions and suspended particulates. This electrochemical process markedly reduces Chemical Oxygen Demand (COD) and Total Suspended Solids (TSS), thereby enhancing the suitability of recovered water for reintegration into industrial processes.

### 5.2. Biological Methods for Water Pollution Mitigation in the Cement Industry

While physico-chemical treatment technologies such as filtration, membrane separation, and electrocoagulation are effective in reducing particulate loads and inorganic contaminants, they are often insufficient for the complete removal of dissolved organic compounds and residual nutrients [149]. Consequently, biological purification processes have emerged as a critical complementary strategy for achieving higher effluent quality and long-term environmental compliance. By leveraging microbial metabolism to degrade organic pollutants and stabilize residual contaminants, biological treatment systems provide an additional layer of purification that enhances effluent resilience and supports sustainable water reuse within cement manufacturing operations. Although many biological treatments have lower removal efficiencies for heavy metals compared to mechanical filters, they work remarkably well to degrade the organic material from cement plants [150,151].

Several studies have examined the use of activated sludge processes, as well as hybrid bio-carrier systems, as possible biological means for the reduction in pollution in the cement making process; though, at this point, there is no substantial empirical data available to illustrate the positive impact that either method has at various Cement Manufacturing facilities. Specially designed systems, such as Integrated Fixed-Film Activated Sludge (IFAS) processes, can improve the effectiveness of these biological treatments. Ali et al. [152] compared the performance of natural *Luffa* sponges and synthetic polyurethane (PU) sponges as bio-supporting media in a pilot-scale system consisting of initial settling (primary sedimentation), biological oxidation (aeration), and final settling (settlement) stages. Their findings show that the hybrid use of both media resulted in a better removal efficiency compared to the use of each method. Removal efficiencies of the hybrid configuration were found to be 94.5% for Total Suspended Solids (TSS), 87.8% for Chemical Oxygen Demand (COD), and 90.8% for Biochemical Oxygen Demand (BOD). The improved performance of the Luffa sponge was achieved through chemical pre-treatment, which increased its surface area and allowed more microorganisms to attach to it, leading to higher organic matter removal rates compared to synthetic sponges.

Al-Shukaili [22] emphasized the important role of activated sludge systems in the degradation of organic pollutants, as they have been implemented to promote sustainable water recycling throughout the entire production cycle. They have shown that the biological treatment methods are effective in removing most of the suspended solids and depositing the organic-based waste into the water. Although there are positive results, the implementation of activated sludge and hybrid systems is hindered due to the limited number of large-scale experimental sites in a wide range of industries.

The ultimate standard for wastewater management in the cement sector is the implementation of Zero Liquid Discharge (ZLD) systems, which integrate advanced technologies such as reverse osmosis, evaporation, and crystallization to eliminate the effluent discharge. By treating and circulating all process water within a closed loop, ZLD provides a comprehensive solution to aquatic pollution, particularly in arid regions where freshwater conservation is critical [153,154,155,156,157]. Despite the high capital costs and substantial energy requirements associated with operating ZLD systems, they remain a promising option for eliminating wastewater discharge in the industrial sector. However, their widespread adoption remains limited due to accessibility and economic constraints. Therefore, achieving a sustainable hydrological profile in cement manufacturing will require not just technological innovation, but also economic and policy frameworks to provide the necessary support [6,157].

## 6. Policy and Regulatory Measures to Reduce Pollution from Cement Manufacturing

Effective mitigation of pollution from cement manufacturing requires not only technological innovation but also robust policy and regulatory frameworks. Policy and regulatory measures play a critical role in setting enforceable standards, guiding industry practices, and ensuring accountability across all stages of cement production. This section examines the regulatory approaches designed to reduce the environmental impacts of cement manufacturing, with particular emphasis on measures addressing carbonation and greenhouse gas emissions, noise pollution, and the prevention of soil and water contamination. Through the integration of emissions limits, land and water protection regulations, and compliance mechanisms, these policies serve as essential tools for minimizing the industry’s environmental footprint while promoting sustainable and responsible production.

### 6.1. Regulatory Frameworks and Carbon Economics

The transition toward a low-carbon cement industry is heavily contingent upon robust governmental regulations and proactive policy interventions. Stricter emission standards have been identified as a primary catalyst for industrial decarbonization [158,159].

Utilizing a System of Systems (SoS) methodology, Jokar and Mokhtaram [160] evaluated how shifting production variables (e.g., clinker substitution and the adoption of alternative fuels) could significantly reduce total CO_2_ emissions by 2034. Their analysis suggests that while WHR provides clear economic benefits, its impact on total CO_2_ mitigation is limited compared to big structural changes like clinker substitution. Consequently, they advocate for targeted government support through low-interest loans, research grants, and the implementation of carbon pricing to accelerate the adoption of sustainable practices. Subsequent research [161,162,163] has reinforced the conclusion that targeted financial support mechanisms are critical for overcoming the high upfront capital costs associated with Carbon Capture, Utilization, and Storage (CCUS) deployment in the cement industry. In particular, capital assistance in the form of direct subsidies during the early project development and construction phases has been shown to significantly reduce financial risk and ease the investment burden faced by cement producers. In parallel, emissions trading schemes introduce a market-based price signal for carbon emissions, thereby creating an economic incentive for firms to invest in emissions reduction technologies, including CCUS. By internalizing the cost of carbon, such mechanisms improve the relative competitiveness of low-emission cement production pathways. Furthermore, the availability of targeted low-interest loans and preferential financing instruments has been identified as a key driver for accelerating CCUS adoption, especially in developing and emerging markets where access to capital is limited and financing costs are substantially higher. Collectively, these policy tools—subsidies, carbon pricing, and concessional financing—form an integrated financial framework capable of reducing investment barriers and enabling the broader deployment of CCUS technologies across the global cement sector.

Frameworks such as the EU Industrial Emissions Directive and the US EPA Clean Air Act have set rigorous benchmarks for CO_2_, NO_x_, SO_2_, and PM from cement manufacturing [164,165]. However, as cement production continues to expand rapidly in developing economies, a significant regulatory gap persists. Many of these regions lack the enforcement mechanisms necessary to meet international standards, highlighting the urgent need for a unified global framework [166]. Additionally, carbon price mechanisms, such as carbon taxes and cap-and-trade systems, provide financial incentives for cement manufacturers to reduce emissions by increasing the perception of cost associated with carbon emissions [167,168]. So far, developed countries like the United States and the European Union have implemented some of the best practices in CCUS and enforced strong emission regulations. However, developing countries lack both the financial and physical infrastructure necessary to implement CCUS technologies. In addition, developing countries lack the ability to utilize and maintain CCUS technologies because they do not possess the necessary expertise. To help developing countries meet their global emission reduction targets through the large-scale implementation of CCUS systems, international transfers of technology and international aid programs need to be developed.

Liu et al. [169] examined the impact of policy instruments on the adoption of Low-Carbon Technologies (LCTs) in China. Their analysis revealed that while Waste Heat Recovery (WHR) is currently the most widely implemented LCT, Energy Management and Optimization Systems (EMOS) offer greater potential for systemic energy and carbon reductions across the cement sector. Specifically, the study demonstrated that the introduction of a carbon price of 60 Yuan/t-CO_2_ significantly increased EMOS adoption, and projected that a carbon price of 100 Yuan/t-CO_2_ could drive universal adoption among the surveyed firms by 2025. The authors identified carbon pricing as the principal driver for LCT deployment in China, highlighting its effectiveness in incentivizing low-carbon investments. They also noted that many developing countries face financial and technical barriers that limit the widespread implementation of LCTs. To address these constraints, Liu et al. proposed targeted policy interventions, including subsidies to support investment in Carbon Capture, Utilization, and Storage (CCUS) technologies and facilitating access to global carbon trading markets, as mechanisms to accelerate LCT adoption in resource-constrained contexts.

The global nature of the cement industry necessitates coordinated action among policymakers across countries. In the absence of harmonized emission reduction targets and carbon pricing mechanisms, the sector faces the risk of “carbon leakage,” whereby production relocates to regions with less stringent environmental regulations, undermining the effectiveness of local climate policies. Establishing a level playing field through internationally aligned standards is therefore critical to ensure that the costs associated with sustainable innovation and low-carbon technologies do not compromise the competitiveness of firms operating in highly regulated jurisdictions. Such coordinated frameworks can help drive consistent adoption of best practices, incentivize investment in low-carbon solutions, and prevent regulatory arbitrage that would otherwise hinder global decarbonization efforts [170,171].

### 6.2. Regulatory Frameworks and Policy Measures for Noise Reduction

The effective mitigation of industrial noise in cement manufacturing relies on governments and international organizations establishing, implementing, and enforcing standardized acoustic limits. Policies that integrate financial incentives—such as subsidies, tax breaks, and carbon trading credits—can encourage plants to adopt Best Available Practices (BAPs) and noise-reduction technologies, making compliance both economically and environmentally advantageous. By linking noise control measures with broader climate goals, cement producers can simultaneously reduce greenhouse gas emissions while improving operational sound management.

Comprehensive noise control regulations must clearly define permissible sound levels for both industrial and residential areas [172]. Establishing such ‘noise contours’ helps prevent the encroachment of residential developments into high-decibel industrial zones, thereby mitigating public health risks. Many national and regional authorities reference the World Health Organization (WHO) Guidelines for Community Noise, which provide evidence-based thresholds to minimize annoyance and sleep disruption. Similarly, the European Union’s Industrial Emissions Directive (IED) mandates the use of Best Available Techniques (BAT) to reduce noise emissions at the source, ensuring that cement facilities comply with stringent environmental standards [7]. Effective enforcement, including regular monitoring and acoustic auditing, is critical; without consistent oversight, technological and administrative improvements in noise control may remain underutilized.

The transition from policy to practice requires robust regulatory oversight. Mohamad et al. [29] emphasize that governments should go beyond exposure limits by mandating BAP adoption. Financial mechanisms, including carbon pricing, carbon trading programs, and targeted subsidies, can incentivize plants to reduce both noise and carbon emissions. In developing countries, where capital constraints may limit the adoption of noise-reduction technologies, low-interest loans and other financial support programs can facilitate investment in acoustic barriers, equipment upgrades, and maintenance protocols to prevent mechanical degradation that exacerbates noise. Continuous monitoring is essential to ensure compliance.

Supporting this, Hadzi-Nikolova et al. [116] highlight that enforcement is ineffective without continuous monitoring programs. By deploying localized sensors both within the facility and at the perimeter, operators can generate real-time data to verify compliance with zoning laws. These acoustic audits serve as a critical diagnostic tool, allowing engineers to pinpoint ‘nuisance noise’ sources that might otherwise be overlooked during general plant inspections.

Regulatory compliance is further reinforced by social and economic incentives. Alsop [173] highlights that fines and penalties create tangible motivations for plants to invest in noise reduction. Moreover, collaborative governance, engaging residents in transparent feedback loops, can help build community trust and acceptance to operate, allowing noise mitigation strategies to be tailored to local needs and reducing the risk of disputes or litigation.

The success of noise-reduction policies is closely tied to enforcement rigor and monitoring frequency. Coordinated international approaches, including harmonized carbon pricing and noise standards, would create a level global playing field, reducing the risk of “regulatory arbitrage” where production shifts to regions with weaker requirements. Such unified frameworks benefit developing country cement producers by providing consistent market conditions and supporting competitiveness while advancing both local and global environmental objectives.

Despite these efforts, rapid industrialization in many regions has left the regulatory landscape fragmented, with standards that are technically inadequate or poorly enforced [116,172]. To bridge the gap between policy goals and operational realities, internationalized auditing protocols, automated monitoring, and remote sensing technologies are essential. These measures ensure that cement facilities worldwide meet both environmental and social obligations, translating policy frameworks into effective noise control practices across the industry.

### 6.3. Regulatory Framework and Policy Measures to Reduce Soil Pollution

The mitigation of soil pollution within the cement industry is fundamentally dependent on the stringency of governmental oversight regarding the handling and disposal of hazardous residuals. In addition to having forms of government regulation in place, there are also several financial incentives that could support the use of soil remediating technologies by helping to subsidise some of the initial investment costs to be incurred (e.g., phytoremediation and bioremediation) and the use of carbon-trading systems. Aside from providing regulatory oversight, governments could provide tax benefits to companies that implement alternative fuel use or minimize their impact on soil contamination through low-impact processes or disposal methods.

To prevent the leaching of CKD and heavy metals into the pedosphere, it is essential that regulatory bodies mandate comprehensive waste management protocols that treat these by-products as controlled substances [142,174]. International benchmarks, such as the EU Industrial Emissions Directive (IED) and U.S. EPA standards [175,176], provide a robust template for this by-law, requiring facilities to adhere to strict limits for pollutants released into the air, land, and water.

The efficacy of these multi-layered regulations is further demonstrated by Juarez and Finnegan [177], who analyzed the intersection of the IED and the EU Emissions Trading Scheme (EU ETS). Their case study of a cement facility revealed that compliance with these dual frameworks led to a significant ‘co-benefit’ effect, resulting in a 65% reduction in SO_2_, a 30% reduction in NO_x_, and an 80% reduction in PM emissions. Since atmospheric particulates serve as the primary vector for heavy metal deposition, these reductions in air emissions are directly correlated to a decreased risk of soil contamination. However, effective governance extends beyond the establishment of limits and requires a proactive infrastructure for consistent environmental auditing.

Yi et al. [178] emphasize the necessity of routine soil and water sampling to identify contaminant “hot spots” before they evolve into systemic ecological crises. To ensure the integrity of these monitoring systems, government agencies must prioritize funding for automated tracking technologies and encourage collaborative governance through community participation in reporting violations [20]. Governments can also help by providing financial support for adopting new environmentally friendly methods of cleaning up contaminated soils and funding local environmental monitoring efforts. When governments work with local communities, they create an opportunity for citizens to report on soil contamination and provide economic benefits to those who implement low-cost ways to clean up their local soils.

Despite the availability of these frameworks, a global “regulatory void” persists in many high-growth regions where enforcement mechanisms remain inadequate, allowing facilities to operate without the oversight necessary to protect local land resources and public health. International cooperation in establishing regulatory frameworks pertaining to soil pollution controls and carbon pricing is necessary to fill the need for regulations in rapidly growing areas where a lack of established regulations exists. Through technology exchange programs and financial assistance provided by more developed countries, Developing Countries can learn how to implement best practices in soil remediation. This will ensure that cement manufacturing facilities in these Developing Countries comply with the same global standards applied to all other facilities for controlling soil pollution.

### 6.4. Regulatory Framework and Policy Measures to Reduce Water Pollution

The mitigation of aquatic degradation in the cement industry is fundamentally driven by the efficacy of governmental oversight and the implementation of standardized effluent protocols. Regulatory bodies serve a critical dual role: defining rigorous benchmarks for wastewater discharge and incentivizing the adoption of circular water management practices within industrial operations.

Economic instruments, including subsidies, tax breaks, and regulatory incentives, can provide the financial support necessary for cement manufacturers to install advanced wastewater treatment and recycling technologies such as Zero Liquid Discharge (ZLD), Reverse Osmosis (RO), and electrocoagulation. By lowering the upfront costs associated with these technologies, such incentives enable broader industry adoption and more sustainable water management practices.

Salman [146] highlights the importance of regulatory oversight through a comparative analysis of Jordanian cement plants, showing that Cementra Jordan operates with an unstructured water management strategy, whereas Lafarge Cement Jordan has implemented systematic internal reuse protocols. This disparity underscores the need for policies that both mandate wastewater treatment and create economic incentives for structured water management. Subsidies for companies that implement water recycling systems or advanced treatment technologies can reduce freshwater consumption, while mandatory regulatory requirements ensure that best practices are consistently applied across the sector. Integrating waste and water management into production efficiency strategies further enhances the industry’s environmental performance.

Sarc et al. [147] examine the regulatory context of Solid Recovered Fuels (SRFs) in Croatia, demonstrating that co-incineration of waste in cement kilns can reduce environmental externalities. Compliance with European Union standards for SRF quality ensures that energy recovery processes are environmentally responsible, illustrating the link between solid waste regulation and lower water pollution. Similarly, Al-Shukaili et al. [22] emphasize the importance of stringent effluent standards for Chemical Oxygen Demand (COD), Biochemical Oxygen Demand (BOD), and Total Suspended Solids (TSS), highlighting Life Cycle Assessment (LCA) as a vital tool for environmental policy and auditing. However, inconsistent enforcement and insufficient financial incentives continue to hinder progress.

#### Bio-Monitoring of Water Pollution: Microbial Toxicity Tests and Ecotoxicological Approaches

Monitoring the effects of pollutants on aquatic ecosystems through bio-monitoring is one of the best ways to determine the health of these environments. As our society continues to produce higher amounts of pollutants due to increasing numbers of industrial, agricultural, and domestic activities, the demand for bio-monitoring water pollution continues to grow. In particular, through various bio-monitoring methods, microbial toxicity testing has been particularly effective in evaluating and quantifying chemical toxins present in aquatic environments using ISO and OECD standardized methodology. These methodologies allow scientists to accurately assess ecosystem health, as well as ensure the safety and quality of water resources used for human consumption.

Strotmann et al. [179] reviewed the ISO/OECD tests that are available for assessing the impact of chemicals on the microbial community and the surrounding aquatic environment. In particular, they provide a description of the sensitivity and limits of the available tests, including respiration inhibition, luminescent bacteria, and inhibition of nitrification. In addition, they introduced a new term (“Physiological Potential of Inoculum”) as a basis for determining whether or not a microbe would make a good inoculum. They recommend that these tests be used as pre-tests to biodegradation studies, and they support the development of an integrated toolbox with the goal of reducing animal testing.

Araujo et al. [180] assessed the Microtox test system to evaluate how much toxicity was removed from industrial effluents collected from the city of Camaçari (Bahia, Brazil). They used the Vibrio fischeri luminescence inhibition method and showed that the toxicity was reduced by 92.71%, and the chemical oxygen demand (COD) decreased by 83.04% after the effluent treatment. They concluded that the Microtox test system is effective for monitoring effluent ecotoxicity and correlating its reduction with decreases in COD.

Microbial toxicity tests are fast, sensitive, and effective for detecting a wide range of toxic effects on microorganisms, including pollutants from industrial waste, wastewater treatment plants, and contaminated water. Among these, assays using luminescent bacteria such as *Vibrio fischeri*—known as Microtox—measure light production to assess the toxicity of pollutants and their impact on microorganisms involved in biological decomposition and nutrient cycling [181,182]. Alba et al. [183] employed the Microtox bioassay to evaluate the toxigenic potential of *Aspergillus fumigatus* isolates from patients with invasive aspergillosis, aspergillomas, colonized individuals, and environmental samples. Toxic culture filtrates examined with luminescent *V. fischeri* revealed significant differences in agglutination ratios, with isolates from invasive aspergillosis patients exhibiting higher toxigenicity. While primarily focused on fungal toxigenicity, the study also demonstrated that the Microtox bioassay is a valuable tool for monitoring environmental contaminants such as mycotoxins in water and treated wastewater systems.

Pagga et al. [184] studied the inhibitory effects of various compounds—N-methylaniline, DMPP, pyrazole, and phenol—on nitrification. The extent of inhibition was closely linked to the compounds’ biodegradability: biodegradable substances like N-methylaniline and phenol caused short-term, reversible inhibition, whereas poorly biodegradable compounds such as DMPP and pyrazole led to prolonged inhibition even at low concentrations. The researchers also highlighted that both the duration and method of chemical application—whether added directly to wastewater or present in a solvent—significantly influence nitrification inhibition. Non-biodegradable toxicants can cause persistent effects, posing substantial risks to wastewater treatment processes.

Biomonitoring often employs activated sludge or pure bacterial cultures, such as *Pseudomonas putida*, to assess pollutant effects on microbial growth. Growth inhibition tests provide insight into microbial population health and the potential long-term impacts of chemical exposure on organisms critical for water treatment [185]. Ecotoxicological assays using higher-trophic-level organisms, including algae, *Daphnia*, and fish, are frequently combined with microbial toxicity tests. This integrated approach allows a comprehensive assessment of pollutants’ effects across multiple organisms and ecosystem functions, revealing both immediate toxicity and potential long-term impacts on biodiversity and ecosystem services [186].

To support monitoring and management efforts, data on the toxicity of cement products have been compiled into summary tables, providing an overview of study types, biological test systems, target organisms, exposure routes, ecotoxicological effects, and human health indicators. Table 2, Table 3 and Table 4 synthesize this information, highlighting the range of microbial and ecotoxicological assays, species tested, and exposure pathways used to assess environmental and human health impacts.

Effluents and waste from cementitious materials are specifically tested for toxic substances, such as heavy metals and highly alkaline compounds, that may harm microbial communities. Microbial toxicity assays, including growth inhibition and luminescent bacteria tests (e.g., Microtox), allow for systematic assessment of these contaminants, enabling comparative evaluation across ecological compartments and human exposure pathways. By integrating bio-monitoring with advanced treatment and recycling policies, the cement industry can both prevent contamination and track the effectiveness of mitigation strategies in real time.

Table 3 contains ecotoxicological data on both aquatic and terrestrial organisms, for example: *Daphnia magna* (Water flea), and Algae: *Chlorella*. The data within this table shows the many varied ways in which concrete contaminates ecosystem health (i.e., Growth inhibition of aquatic plants, behavioral changes/responses of fish).

Table 4 highlights the potential human health risks associated with pollution from cement manufacturing operations. It includes information on the various ways (or ‘pathways’) humans are exposed to cement manufacturing emissions, how these emissions can cause harm to humans (e.g., through inhalation of particulate matter and/or exposure to ‘ambient air pollution’ or ‘dust’ generated from/within residential areas/products derived from cement plant operations), and the associated health risks (e.g., respiratory stress, cardiovascular disease, and long-term effects, e.g., cancer), therefore stressing the need for improved environmental and health monitoring in cement plant facilities.

Microbiological Toxicology Tests (MTT) play an important role in evaluating the environmental impact of water pollution due to their speed, ease of use, and high sensitivity. When combined with ecotoxicity assays and biodegradation studies, MTTs enable a comprehensive assessment of water pollution and inform effective mitigation strategies. Additionally, by reducing the need for animal testing, they offer an ethical and efficient approach to evaluating environmental risks.

In summary, the transition toward sustainable water management requires modernized policy frameworks, robust compliance monitoring, and targeted financial support. Governments should provide subsidies to promote advanced wastewater treatment technologies and financially sustain the research and development for cost-effective water treatment and bio-monitoring solutions. Policies should mandate efficient water recycling and reuse while encouraging the adoption of bio-monitoring programs to continuously assess water quality and ecosystem health. These measures will make sustainable water management technologies accessible, particularly in water-scarce regions, helping the cement industry reduce freshwater use, minimize its hydrological footprint, and safeguard environmental and public health.

## 7. Conclusions

Global cement production represents a profound ecological challenge, generating billions of tons of CO_2_ annually while contributing to air and water pollution, soil contamination, and significant acoustic disturbance. As international demand for cement continues to rise, mitigating these multidimensional impacts has become increasingly critical. Current mitigation strategies primarily emphasize Carbon Capture, Utilization, and Storage (CCUS), the integration of alternative fuels such as biomass, and the development of cleaner production methodologies. Although CCUS and fuel substitution offer viable pathways for decarbonization, their large-scale deployment remains constrained by high capital costs, challenges associated with global scalability, and the inherent risks of early-stage technological implementation.

Beyond carbon emissions, the remediation of localized environmental impacts requires specialized technological approaches, many of which remain limited in scope or applicability. For example, vibration isolation and phytoremediation provide targeted solutions for noise mitigation and soil contamination, respectively, but their effectiveness is often constrained by site-specific geological and operational conditions. Similarly, wastewater generated by cement production—frequently characterized by elevated alkalinity, chemical additives, and heavy metal content—necessitates advanced treatment trains such as filtration, reverse osmosis, and Zero Liquid Discharge (ZLD) systems. Despite their effectiveness, these technologies currently represent only partial solutions due to their high energy demands and substantial implementation costs.

Future development of CCUS technologies should prioritize improvements in efficiency, scalability, and integration with complementary mitigation strategies. This includes the development of lower-energy solvents for CO_2_ capture, cost-effective capture processes, and enhanced utilization of biomass and alternative fuels to stabilize emissions profiles. In parallel, further research is required to advance long-term monitoring systems for geological storage sites to verify containment integrity and assess the risks of leakage over extended timescales. The integration of CCUS with fuel substitution and other emission reduction technologies into unified, cost-effective systems remains essential for global applicability.

Noise pollution associated with cement manufacturing also warrants further investigation, particularly regarding its long-term effects on worker health and surrounding communities. Future research should address potential links to chronic stress-related disorders and cognitive impairment, supported by advanced noise modeling to better characterize exposure patterns beyond hearing loss alone. In addition, systematic cost–benefit and hazard analyses of noise-control measures—such as acoustic barriers and vibration isolation systems—are needed to identify solutions tailored to specific noise-generating processes. Similarly, the impacts of cement-related emissions on agricultural land and food security require closer examination, particularly concerning heavy metal accumulation and long-term soil degradation, alongside the development of large-scale soil remediation and stabilization strategies.

Ultimately, the transition toward a more sustainable cement industry depends on robust regulatory and economic frameworks. Governments must enforce stringent emissions standards, provide targeted financial incentives, and implement carbon pricing mechanisms that reflect the true environmental costs of cement production. Regulatory harmonization and the phased removal of hazardous materials are also essential to support industrial modernization. Achieving global sustainability targets will require sustained investment in research and development, as well as strong cross-sector collaboration between governments and industry, to bridge the gap between technological potential and commercial viability.

## Figures and Tables

**Table 1:** Main pollutants from cement plants and their primary sources.

Stage of Cement Production	Main Activities	Pollution Type	Major Pollutants/Impacts
1. Quarrying & Mining	Limestone extraction, blasting	Air, Noise, Soil, Water	Dust (PM), noise, vibration, and landscape degradation. Suspended solids in runoff, disruption of groundwater tables.
2. Crushing & Grinding (Raw Materials)	Crushing limestone, raw milling	Air, Noise, Water	Particulate matter (cement dust), noise; Contaminated wastewater from equipment cooling and dust suppression.
3. Raw Meal Blending & Storage	Mixing and storage of raw materials	Air	Fugitive dust emissions
4. Preheating & Calcination	Heating raw meal, CO_2_ release	Air, Water	CO_2_, NO_x_, SO_2_, PM; Thermal pollution if cooling water is discharged into local streams
5. Clinker Production (Kiln)	High-temperature kiln operation	Air	CO_2_, NO_x_, SO_2_, CO, particulate matter
6. Clinker Cooling	Cooling hot clinker	Air, Noise	Dust, hot air emissions, noise, and alkaline runoff (high pH) from storage areas.
7. Cement Grinding	Grinding clinker with gypsum	Air, Noise	Fine particulate matter (PM_2_._5_, PM_10_), noise
8. Storage and Packing	Silo storage, bagging	Air, Water	Cement dust, fugitive emissions, and highly alkaline wastewater (pH > 11) from facility wash-downs
9. Transportation	Material handling, truck movement	Air, Noise, Water	Dust, exhaust gases (NO_x_, CO); oil/fuel spills and sediment runoff from transit roads.

**Table 2:** Summary of microbial toxicity assays applied to cement-related pollutants.

Test Organism/ Assay	Toxicity Endpoint	Pollutants Assessed	Key Findings	Reference
Vibrio fischeri (Microtox^®^ bioluminescence test)	Luminescence inhibition (%)	Cement leachates, heavy metals, alkaline effluents	Rapid and sensitive response to metal-rich and high-pH cement leachates, indicating acute microbial toxicity	[187]
Activated sludge microorganisms	Respiration inhibition	Cement kiln dust (CKD), high alkalinity wastewater	Significant reduction in microbial respiration at high pH and metal concentrations, affecting biological treatment efficiency	[31]
Recombinant luminescent bacteria	Metal bioavailability response	Cd, Zn, Hg in contaminated soils	High sensitivity to bioavailable heavy metals in cement-impacted soils	[188]
Mixed bacterial consortia	Growth inhibition	Cement wastewater	Inhibited microbial growth under alkaline and metal-rich conditions	[189]

**Table 3:** Ecotoxicological effects of cement-related pollution on aquatic and terrestrial organisms.

Organism	Exposure Medium	Toxicity Endpoint	Observed Ecotoxicological Effects	Reference
Daphnia magna	Cement effluent	LC_50_, mobility inhibition	Acute toxicity observed at elevated heavy metal concentrations	[190]
Algae (*Chlorella* sp.)	Surface water near cement plants	Growth inhibition	Reduced photosynthetic activity and biomass growth	[32]
Fish larvae	Contaminated river water	Survival, behavior	Altered swimming behavior and increased mortality	[31]
Agricultural crops	Cement-contaminated soil	Metal uptake (bioaccumulation)	Elevated Pb, Cd, and Cr concentrations in edible plant tissues	[191]
Soil invertebrates	Industrial dust-impacted soil	Survival and reproduction	Reduced population density and soil fertility indicators	[192]

**Table 4:** Human toxicity and health risk indicators associated with cement industry pollution.

Exposure Pathway	Major Pollutants	Health Endpoint	Reported Health Risks	Reference
Occupational inhalation	PM, noise, heavy metals	Hearing loss, respiratory stress	Elevated prevalence of noise-induced hearing loss and respiratory symptoms among workers	[19]
Ambient air exposure	PM, NO_x_, SO_2_	Cardiovascular and pulmonary effects	Increased risk of chronic respiratory diseases in nearby populations	[193]
Drinking water contamination	Heavy metals, high pH	Renal and gastrointestinal effects	Potential long-term health risks due to chronic metal exposure	[32]
Soil–food chain transfer	Pb, Cd, Cr	Food safety and toxicity	Dietary exposure risks due to metal bioaccumulation in crops	[194]
Residential dust exposure	Cr, Ni, Pb	Cancer and non-cancer risk	Higher health risk indices reported for children living near cement plants	[195]

## Data Availability

No new data were created or analyzed in this study. Data sharing is not applicable to this article.
